# Supplementary material for: Simple Disposable Odor Identification Tests for Predicting SARS-CoV-2 Positivity
Source: Int J Environ Res Public Health. 2021 Sep 28;18(19):10185. doi: 10.3390/ijerph181910185 (PMC8507979; doi:10.3390/ijerph181910185)
Supplement: Supplementary file 1 [file ijerph-18-10185-s001.zip › ijerph-1364739-supplementary.pdf]

Supplementary materials

Table S1. The intergroup comparison of objective smell test results for all evaluated odors (Fisher's exact test).

	Cases (n = 64)	Controls (n = 34)	Total (n = 98)	p-Value
		**Detection**		
cinnamon	55 (85.9%)	34 (100%)	89 (90.8%)	0.025
mint	52 (81.3%)	34 (100%)	86 (87.8%)	0.007
lemon	52 (81.3%)	34 (100%)	86 (87.8%)	0.007
coffee	54 (84.4%)	34 (100%)	88 (89.8%)	0.014
clove	54 (84.4%)	34 (100%)	88 (89.8%)	0.014
rose	57 (89.1%)	34 (100%)	91 (92.9%)	0.092
anise	55 (85.9%)	34 (100%)	89 (90.8%)	0.025
camphor	53 (82.8%)	34 (100%)	87 (88.8%)	0.008
alcohol	49 (76.6%)	34 (100%)	83 (84.7%)	0.001
		Identification		
cinnamon	39 (60.9%)	34 (100%)	73 (74.5%)	<0.001
mint	41 (64.1%)	34 (100%)	75 (76.5%)	<0.001
lemon	41 (64.1%)	33 (97.1%)	74 (75.5%)	<0.001
coffee	44 (68.8%)	31 (91.2%)	75 (76.5%)	0.013
clove	46 (71.9%)	33 (97.1%)	79 (80.6%)	0.002
rose	42 (65.6%)	29 (85.3%)	71 (72.4%)	0.056
anise	45 (70.3%)	33 (97.1%)	78 (79.6%)	0.001
camphor	46 (71.9%)	33 (97.1%)	79 (80.6%)	0.002
alcohol	34 (53.1%)	33 (97.1%)	67 (68.4%)	<0.001

Table S2. Detailed correlations between self-reported OD and clinical characteristics of COVID-19 patients.

**Variable**		**Self-Reported OD**					
		**Presence of Self-Reported OD at the Time of the Survey**			**Presence of Self-Reported OD at Any Time since the Onset of COVID-19**		
		**yes (N = 21)**	**no (N = 43)**	**p-Value**	**yes (N = 27)**	**no (N = 37)**	**p-Value**
Nasal congestion, N (%)	yes	11 (52.4)	10 (23.3)	0.041[3]	14 (51.9)	7 (18.9)	0.012 [3]
	no	10 (47.6)	33 (76.7)		13 (48.1)	30 (81.1)	
Rhinorrhea, N (%)	yes	9 (42.9)	10 (23.3)	0.187 [3]	14 (51.9)	5 (13.5)	0.002 [3]
	no	12 (57.1)	33 (76.7)		13 (48.1)	32 (86.5)	
Presence of other symptoms at the time of the survey, N (%)[1]	yes	15 (71.4)	20 (46.5)	0.107 [3]	17 (63)	18 (48.6)	0.378[3]
	no	6 (28.6)	23 (53.5)		10 (37)	19 (51.4)	
Presence of other symptoms since onset of COVID-19, N (%)[2]	yes	21 (100)	34 (79.1)	0.025 [5]	27 (100)	28 (75.7)	0.008 [5]
	no	0 (0)	9 (20.9)		0 (0)	9 (24.3)	
Current smoking, N (%)	yes	3 (14.3)	5 (11.6)	1[5]	3 (11.1)	5 (13.5)	1[5]
	no	18 (85.7)	38 (88.4)		24 (88.9)	32 (86.5)	
Former or current smoking, N (%)	yes	8 (38.1)	21 (48.8)	0.587 [3]	8 (29.6)	21 (56.8)	0.058 [3]
	no	13 (61.9)	22 (51.2)		19 (70.4)	16 (43.2)	
Death, N (%)	yes	2 (9.5)	6 (14)	1 [5]	2 (7.4)	6 (16.2)	0.450 [5]
	no	19 (90.5)	37 (86)		25 (92.6)	31 (83.8)	
Need for oxygen therapy, N (%)	yes	7 (33.3)	21 (48.8)	0.365 [3]	7 (25.9)	21 (56.8)	0.028 [3]
	no	14 (66.7)	22 (51.2)		20 (74.1)	16 (43.2)	
Need for ICU stay, N (%)	yes	1 (4.8)	5 (11.6)	0.654 [5]	1 (3.7)	5 (13.5)	0.388 [5]
	no	20 (95.2)	38 (88.4)		26 (96.3)	32 (86.5)	
Need for invasive ventilation, N (%)	yes	1 (4.8)	4 (9.3)	1 [5]	1 (3.7)	4 (10.8)	0.387 [5]
	no	20 (95.2)	39 (90.7)		26 (96.3)	33 (89.2)	
Number of other symptoms at the time of the survey	Mean ± SD	1.7 ± 1.6	1 ± 1.4	0.057 [4]	1.4 ± 1.5	1.1 ± 1.5	0.284 [4]
	Median (IQR)	1 (0–3)	0 (0–2)		1 (0–2)	0 (0–2)	
Number of other symptoms since the onset of COVID-19	Mean ± SD	4.2 ± 2.2	2.9 ± 2.1	0.040 [4]	4.2 ± 1.9	2.73 ± 2.2	0.013 [4]
	Median (IQR)	4 (3–6)	3 (1–4)		4 (3–5)	3 (1–4)	
Time interval between first positive PCR result and time of the survey, days	Mean ± SD	5.1 ± 4.2	7.6 ± 6.7	0.290 [4]	6.7 ± 4.8	7.1 ± 7	0.615 [4]
	Median (IQR)	3 (2–8)	6 (2–12)		5 (2.5–12)	4 (2–12)	
Duration of infection (from first positive to first negative PCR result, excluding deceased patients)	N	19	37	0.134 [4]	25	31	0.014 [4]
	Mean ± SD	17.47 ± 10.12	21.57 ± 13.14		16.48 ± 9.12	23.16 ± 13.73	
	Median (IQR)	13 (10–21.5)	16 (14–26)		12 (11–17)	18 (14–28.5)	
Duration of hospitalisation (excluding deceased	N	19	37	0.862 [4]	25	31	0.060 [4]

patients)	Mean ± SD	19 ± 10.64	20.68 ± 14.2		16.56 ± 10.5	22.97 ± 14.28	
	Median (IQR)	17 (12–23.5)	18 (10–24)		13 (10–19)	18 (15–28)	
MEWS score at the time of the survey	Mean ± SD	0.86 ± 1.28	0.86 ± 1.67	0.837[4]	0.67 ± 1.18	1 ± 1.76	0.254[4]
	Median (IQR)	0 (0–1)	0 (0–1)		0 (0–1)	1 (0–1)	
Avarage MEWS score	Mean ± SD	0.86 ± 1.49	0.98 ± 1.32	0.481[4]	0.7 ± 1.35	1.11 ± 1.37	0.081[4]
	Median (IQR)	0 (0–1)	1 (0–1)		0 (0–1)	1 (0–2)	

[1] including: fatigue—22 (34.4%), cough—15 (23.4%), fever—2 (3.1%), headache—6 (9.4%), gastrointestinal complaints—14 (21.9%), nasal congestion—5 (7.8%), rhinorhhea—5 (7.8%), chills—2 (3.1%), dyspnea—7 (10.9%). [2] including: fatigue—45 (70.3%), cough—25 (39.1%), fever—24 (37.5%), headache—23 (35.9%), gastrointestinal complaints—23 (35.9%), nasal congestion—21 (32.8%), rhinorhhea—19 (29.7%), chills—18 (28.1%), dyspnea—15 (23.4%). [3] chi-squared test. [4] Mann-Whitney test, [5] Fisher test.

Table S3. Detailed correlations between objective OD (according to SDOIT) and quantitative clinical characteristics of COVID-19 patients; Mann-Whitney test.

**Variable**		**SDOIT, % of Correct Answers**							
		**10-SDOIT**		**9-SDOIT**		**8-SDOIT**		**4-SDOIT**	
		Mean ± SD	Median (IQR)	Mean ± SD	Median (IQR)	Mean ± SD	Median (IQR)	Mean ± SD	Median (IQR)
Nasal congestion	yes	72.9 ± 30.4	90 (50–100)	72 ± 33.5	88.9 (55.6–100)	71.4± 33.1	87.5 (50–100)	70.2 ± 40	100 (25–100)
	no	63 ± 32.3	70 (45–90)	62.5 ± 34.6	77.8 (44.4–88.9)	62.8 ± 34.9	75 (37.5–87.5)	55.8 ± 38.1	75 (25–87.5)
	p-value	0.188		0.197		0.240		0.081	
Rhinorrhea	yes	73.7 ± 30.4	90 (50–100)	73.1 ± 32.4	88.9 (56–100)	73.3 ± 32.1	87.5 (56.3–100)	69.7 ± 37.8	100 (37.5–100)
	no	63.1 ± 32.2	80 (40–90)	62.5 ± 34.9	77.8 (44.4–88.9)	62.5 ± 35.1	75 (37.5–87.5)	56.7 ± 39.3	75 (25–100)
	p-value	0.110		0.152		0.179		0.153	
Presence of other symptoms at the time of the survey	yes	60 ± 31.2	60 (40–90)	59.8 ± 33.9	66.7 (38.9–88.9)	60 ± 33.3	62.5 (37.5–87.5)	55 ± 37.3	50 (25–87.5)
	no	73.8 ± 31.3	90 (50–100)	73.2 ± 33.8	88.9 (55.6–100)	72.4 ± 34.8	87.5 (50–100)	67.2 ± 40.7	75 (25–100)
	p-value	**0.032**		**0.042**		**0.042**		0.139	
Presence of other symptoms since onset of COVID-19	yes	66.7 ± 32.4	80 (50–90)	66.1 ± 35.2	77.8 (55.6–88.9)	66.4 ± 34.9	75 (50–93.8)	62.3 ± 39.1	75 (25–100)
	no	63.3 ± 29.2	50 (40–90)	63 ± 29.4	55.6 (44.4–88.9)	61.1 ± 31.5	62.5 (37.5–87.5)	50 ± 39.5	50 (25–75)
	p-value	0.711		0.617		0.576		0.368	
Current smoking	yes	70 ± 31.2	85 (55–90)	68.1 ± 35.9	83.3 (50–91.7)	68.8 ± 34.7	81.3 (56.4–90.6)	65.6 ± 37.7	75 (43.8–100)
	no	65.7 ± 32.1	80 (47.5–90)	65.3 ± 34.4	77.8 (44.4–88.9)	65.2 ± 34.5	75 (37.5–90.6)	59.8 ± 39.5	75 (25–100)
	p-value	0.806		0.837		0.829		0.745	
Former or current smoking	yes	63.1 ± 30.7	80 (40–90)	62.8 ± 32.8	77.8 (44.4–88.9)	62.9 ± 32.8	75 (37.5–87.5)	55.2 ± 40.3	75 (25–100)
	no	68.9 ± 32.9	80 (50–100)	67.9 ± 35.7	77.8 (55.6–100)	67.9 ± 35.8	75 (56.3–100)	65 ± 38	75 (37.5–100)
	p-value	0.241		0.259		0.308		0.335	
Death	yes	51.2 ± 22.3	60 (55–60)	50 ± 21.4	55.6 (52.8–58.3)	51.6 ± 23.6	62.5 (46.9–62.5)	40.6 ± 29.7	37.5 (25–50)
	no	68.4 ± 32.5	8 (47.5–90)	67.9 ± 35.3	88.9 (44.4–100)	67.6 ± 35.3	87.5 (37.5–100)	63.4 ± 39.6	75 (25–100)
	p-value	0.066		**0.048**		**0.047**		0.108	
Need for oxygen therapy	yes	58.9 ± 30.6	6 (47.5–80)	58.3 ± 32.3	61.1 (52.8–80.6)	58.9 ± 32.4	62.5 (46.9–78.1)	49.1 ± 35.7	50 (18.8–75)
	no	71.9 ± 32	90 (47.5–100)	71.3 ± 35.2	88.9 (44.4–100)	70.8 ± 35.2	87.5 (37.5–100)	69.4 ± 39.7	100 (25–100)
	p-value	**0.032**		**0.026**		**0.035**		**0.013**	
Need for ICU stay	yes	68.3 ± 16	60 (60–67.5)	66.7 ± 17.2	61.1 (55.6–66.7)	68.8 ± 17.2	62.5 (62.5–71.9)	58.3 ± 34.2	50 (31.3–87.5)
	no	66 ± 33.1	80 (40–90)	65.5 ± 35.7	77.8 (44.4–88.9)	65.3 ± 35.7	75 (37.5–96.9)	60.8 ± 39.8	75 (25–100)
	p-value	0.852		0.700		0.682		0.877	
Need for invasive ventilation	yes	62 ± 4.5	60 (60–60)	60 ± 6.09	55.6 (55.6–66.7)	62.5 ± 8.8	62.5 (62.5–62.5)	50 ± 30.6	50 (25–50)
	no	66.9 ± 33.3	80 (40–90)	66.3 ± 35.9	83.3 (44.4–97.2)	65.9 ± 35.7	75 (37.5–100)	61.6 ± 40.1	75 (25–100)

p-value	0.355	0.278	0.292	0.485

Table S4. Detailed correlations between objective OD (according to SDOIT) and qualitative clinical characteristics of COVID-19 patients; Spearman correlation.

**Variable**		**SDOIT**			
		**10-SDOIT**	**9-SDOIT**	**8-SDOIT**	**4-SDOIT**
Number of other symptoms at the time of the survey	ρ	-0.28	-0.27	-0.27	-0.19
	p-value	0.024	0.031	0.033	0.142
Number of other symptoms since the onset of COVID-19	ρ	0.06	0.07	0.09	0.14
	p-value	0.613	0.575	0.486	0.287
Time interval between first positive PCR result and time of the survey, days	ρ	0.19	0.18	0.19	0.1
	p-value	0.123	0.163	0.141	0.447
Duration of infection (from first positive to first negative PCR, excluding deceased)	ρ	-0.15	-0.14	-0.14	-0.11
	p-value	0.265	0.294	0.319	0.422
Duration of hospitalisation (excluding deceased)	ρ	-0.43	-0.42	-0.41	-0.36
	p-value	<0.001	0.002	0.002	0.007
MEWS score at the time of the survey	ρ	-0.29	-0.32	-0.32	-0.25
	p-value	0.02	0.011	0.011	0.043
Avarage MEWS score	ρ	-0.29	-0.3	-0.29	-0.24
	p-value	0.02	0.016	0.018	0.054

Table S5. Correlations between self-reported OD and general characteristics of the entire cohort (cases and controls, *N* = 64).

**Variable**		**Self-Reported OD**					
		**Presence of Self-Reported OD at the Time of the Survey**			**Presence of Self-Reported OD at Any Time since the Onset of COVID-19**		
		**Yes (*N* = 21)**	**No (*N* = 43)**	***p*-Value**	**Yes (*N* = 27)**	**No (*N* = 37)**	***p*-Value**
Age	Mean ± SD	55.1 ± 21.5	46.5 ± 17.7	0.395[1]	47.6 ± 23.7	48.6 ± 16.7	0.583[1]
	Median (IQR)	57 (34–72)	45 (32–60)		45 (24–69.5)	47 (34–62.5)	
Gender, N (%)	female	14 (66.7)	41 (53.2)	0.842[2]	15 (55.6)	40 (56.3)	1[2]
	male	7 (33.3)	36 (46.8)		12 (44.4)	31 (43.7)	
Current smoking	yes	3 (14.3)	5 (11.6)	1[3]	3 (11.1)	5 (13.5)	1[3]
	no	18 (85.7)	38 (88.4)		24 (88.9)	32 (86.5)	
Current or former smoking	yes	8 (38.1)	21 (48.8)	0.587[1]	8 (29.6)	21 (56.8)	0.058[1]
	no	13 (61.9)	22 (51.2)		19 (70.4)	15 (43.2)	

[1]Mann-Whitney test., [2] chi-squared test, [3] Fisher test

Table S6. Correlations between gender and objective OD (according to SDOIT) of the entire cohort (cases and controls, *N* = 64); Mann-Whitney test.

**Variable**		**SDOIT, % of Correct Answers**							
		**10-SDOIT**		**9-SDOIT**		**8-SDOIT**		**4-SDOIT**	
		Mean ± SD	Median (IQR)	Mean ± SD	Median (IQR)	Mean ± SD	Median (IQR)	Mean ± SD	Median (IQR)
**Gender**	female	77.9 ± 30.4	90 (55–100)	74.8 ± 32.4	77.8 ± 30.6	75.3 ± 33.8	78.1 ± 30.9	73.6 ± 36.8	73.8 ± 36.6
	male	78.4 ± 28.9	90 (75–100)	88.9 (55.6–100)	88.9 (72.2–100)	85.7 (71.4–100)	85.7 (71.4–100)	100 (50–100)	100 (50–100)
	p-value	0.641		0.676		0.822		0.978	

Table S7. Detailed results of the ROC analysis.

**Classifier**	**Sensitivity**	**Specifity**	**PPV**	**NPV**	**AUC**
Self-reported OD at the time of the survey	0.33 (CI95% 0.22–0.45)	1 (CI95% 1–1)	1 (CI95% 1–1)	0.44 (CI95% 0.4–0.49)	0.66 (CI95% 0.61–0.72)
Self-reported OD at the time of maximum deterioration	0.42 (CI95% 0.3–0.55)	1 (CI95% 1–1)	1 (CI95% 1–1)	0.48 (CI95% 0.43–0.54)	0.71 (CI95% 0.65–0.77)
Maximum VAS	0.64 (CI95% 0.53–0.75)	1 (CI95% 1–1)	1 (CI95% 1–1)	0.6 (CI95% 0.53–0.68)	0.82 (CI95% 0.76–0.88)
10-SDOIT (cut-off at 95% - OD ≥ 1 incorrect)	0.8 (CI95% 0.56–0.78)	0.71 (CI95% 0.62–0.76)	0.84 (CI95% 0.79–0.86)	0.65 (CI95% 0.52–0.77)	0.82 (CI95% 0.74–0.9)
10-SDOIT (OD ≥ 1 incorrect) + self-	0.91 (CI95% 0.83–0.97)	0.71 (CI95% 0.56–0.85)	0.85 (CI95% 0.79–0.92)	0.8 (CI95% 0.67–0.93)	0.87 (CI95% 0.8–0.93)

reported OD					
10-SDOIT (OD ≥2 incorrect)	0.59 (CI95% 0.47–0.72)	0.91 (CI95% 0.82–1)	0.93 (CI95% 0.85–1)	0.54 (CI95% 0.47–0.63)	0.75 (CI95% 0.68–0.83)
10-SDOIT (OD OD ≥2 incorrect) + self-reported OD	0.77 (CI95% 0.66–0.86)	0.91 (CI95% 0.82–1)	0.94 (CI95% 0.88–1)	0.67 (CI95% 0.58–0.78)	0.86 (CI95% 0.80–0.92)
9-SDOIT (cut-off at 94% - OD ≥ 1 incorrect)	0.77 (CI95% 0.55–0.86)	0.71 (CI95% 0.59–0.97)	0.83 (CI95% 0.77–0.97)	0.62 (CI95% 0.5–0.74)	0.80 (CI95% 0.73–0.88)
9-SDOIT (OD ≥ 1 incorrect) + self-reported OD	0.88 (CI95% 0.83–0.97)	0.71 (CI95% 0.56–0.85)	0.85 (CI955 0.79–0.92)	0.75 (CI95% 0.68–0.93)	0.85 (CI95% 0.79–0.92)
9-SDOIT (OD ≥2 incorrect)	0.55 (CI95% 0.42–0.67)	0.94 (CI95% 0.85–1)	0.95 (CI95% 0.87–1)	0.52 (CI95% 0.46–0.6)	0.74 (CI95% 0.67–0.82)
9-SDOIT (OD OD ≥2 incorrect) + self-reported OD	0.73 (CI95% 0.83–0.97)	0.94 (CI95% 0.56–0.85)	0.96 (CI95% 0.79–0.92)	0.65 (CI95% 0.67–0.93)	0.85 (CI95% 0.79–0.91)
8-SDOIT (cut-off at 94% - OD ≥ 1 incorrect)	0.75 (CI95% 0.61–0.86)	0.79 (CI95% 0.68–0.94)	0.87 (CI95% 0.81–0.96)	0.63 (CI95% 0.53–0.74)	0.82 (CI95% 0.75–0.89)
8-SDOIT (OD ≥ 1 incorrect) + self-reported OD	0.86 (CI95% 0.77–0.94)	0.79 (CI95% 0.65–0.91)	0.89 (CI95% 0.82–0.95)	0.75 (CI95% 0.64–0.88)	0.87 (CI95% 0.81–0.93)
8-SDOIT (OD ≥2 incorrect)	0.55 (CI95% 0.42–0.67)	0.97 (CI95% 0.91–1)	0.97 (CI95% 0.91–1)	0.53 (CI95% 0.47–0.61)	0.76 (CI95% 0.69–0.83)
8-SDOIT (OD OD ≥2 incorrect) + self-reported OD	0.73 (CI95% 0.62–0.84)	0.97 (CI95% 0.91–1)	0.98 (CI95% 0.93–1)	0.66 (CI95% 0.58–0.76)	0.86 (CI95% 0.8–0.92)
4-SDOIT (cut-off at 88% - ≥ 1 incorrect)	0.64 (CI95% 0.53–0.75)	0.94 (CI95% 0.85–1)	0.95 (CI95% 0.89–1)	0.58 (CI95% 0.51–0.67)	0.80 (CI95% 0.74–0.87)
4-SDOIT (OD ≥ 1 incorrect) + self-reported OD	0.78 (CI95% 0.67–0.88)	0.94 (C I95% 0.85–1)	0.96 (CI95% 0.91–1)	0.7 (CI95% 0.6–0.8)	0.87 (CI95% 0.82–0.93)
4-SDOIT (OD ≥2 incorrect)	0.42 (CI95% 0.3–0.53)	1 (CI95% 1–1)	1 (CI95% 1–1)	0.48 (CI95% 0.43–0.53)	0.72 (CI95% 0.65–0.77)
4-SDOIT (OD ≥2 incorrect) + self-reported OD	0.64 (CI95% 0.52–0.75)	1 (CI95% 1–1)	1 (CI95% 1–1)	0.6 (CI95% 0.52–0.68)	0.82 (CI95% 0.76–0.88)

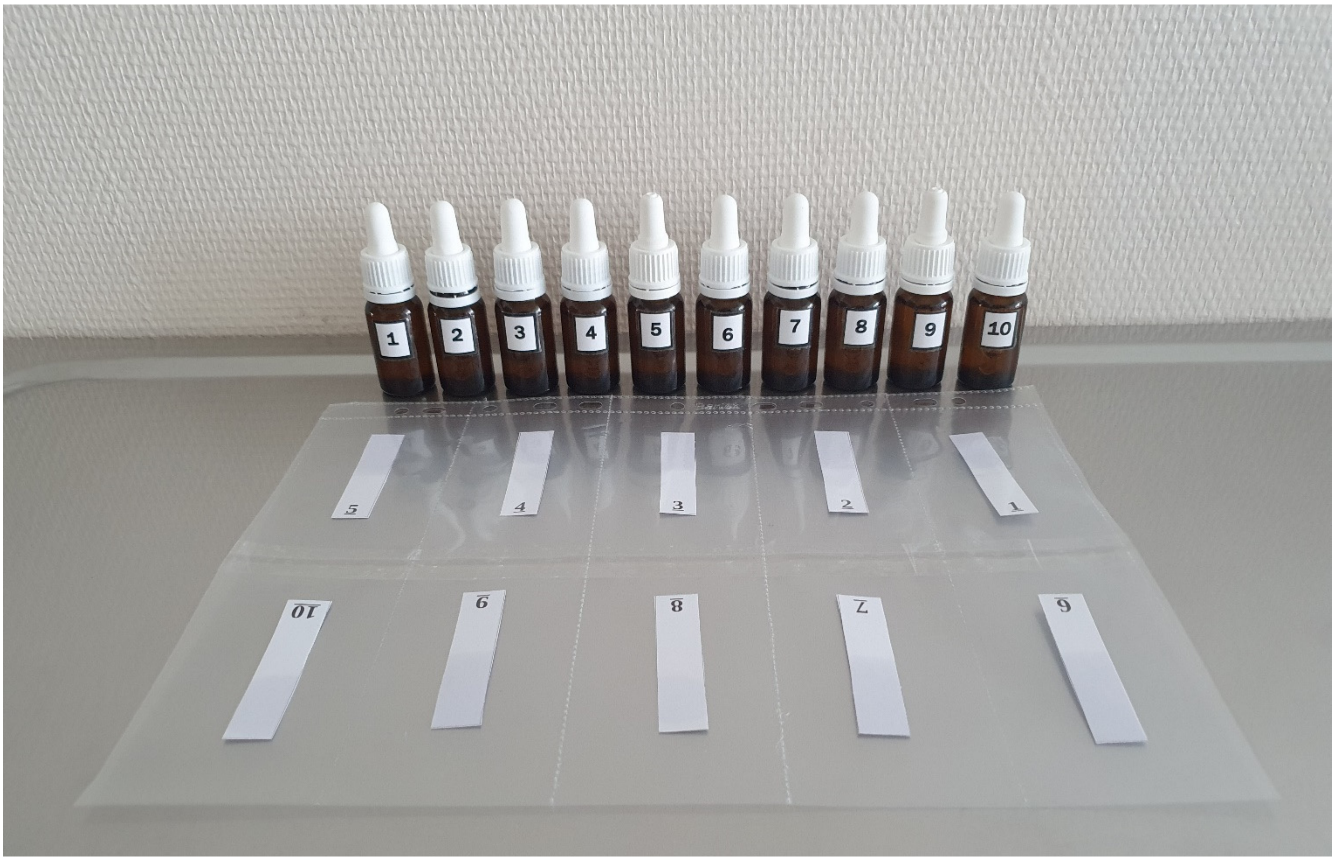

Figure S1. SDOIT test kit example: (**A**) 10 disposable test paper strips numbered 1 to 10 enclosed in plastic covers; (**B**) Odorants (commercially available cinnamon, mint, lemon, coffee, clove, rose, anise and camphor oil, and deionaised water, numbered 1 to 10) in dropper bottles.
